# Reductions in External Divalent Cations Evoke Novel Voltage-Gated Currents in Sensory Neurons

**DOI:** 10.1371/journal.pone.0031585

**Published:** 2012-02-06

**Authors:** Parmvir K. Bahia, Eric S. Bennett, Thomas E. Taylor-Clark

**Affiliations:** Department of Molecular Pharmacology and Physiology, College of Medicine, University of South Florida, Tampa, Florida, United States of America; Dalhousie University, Canada

## Abstract

It has long been recognized that divalent cations modulate cell excitability. Sensory nerve excitability is of critical importance to peripheral diseases associated with pain, sensory dysfunction and evoked reflexes. Thus we have studied the role these cations play on dissociated sensory nerve activity. Withdrawal of both Mg^2+^ and Ca^2+^ from external solutions activates over 90% of dissociated mouse sensory neurons. Imaging studies demonstrate a Na^+^ influx that then causes depolarization-mediated activation of voltage-gated Ca^2+^ channels (Ca_V_), which allows Ca^2+^ influx upon divalent re-introduction. Inhibition of Ca_V_ (ω-conotoxin, nifedipine) or Na_V_ (tetrodotoxin, lidocaine) fails to reduce the Na^+^ influx. The Ca^2+^ influx is inhibited by Ca_V_ inhibitors but not by TRPM7 inhibition (spermine) or store-operated channel inhibition (SKF96365). Withdrawal of either Mg^2+^ or Ca^2+^ alone fails to evoke cation influxes in vagal sensory neurons. In electrophysiological studies of dissociated mouse vagal sensory neurons, withdrawal of both Mg^2+^ and Ca^2+^ from external solutions evokes a large slowly-inactivating voltage-gated current (*I_DF_*) that cannot be accounted for by an increased negative surface potential. Withdrawal of Ca^2+^ alone fails to evoke *I_DF_*. Evidence suggests *I_DF_* is a non-selective cation current. The *I_DF_* is not reduced by inhibition of Na_V_ (lidocaine, riluzole), Ca_V_ (cilnidipine, nifedipine), K_V_ (tetraethylammonium, 4-aminopyridine) or TRPM7 channels (spermine). In summary, sensory neurons express a novel voltage-gated cation channel that is inhibited by external Ca^2+^ (IC_50_∼0.5 µM) or Mg^2+^ (IC_50_∼3 µM). Activation of this putative channel evokes substantial cation fluxes in sensory neurons.

## Introduction

Sensory vagal afferent nerves express specific ion channels that are gated by mechanical, thermal, osmotic, acid/base and chemical stimuli; activation of these ion channels leads to membrane depolarization and the initiation and propagation of action potentials centrally towards the CNS [1]. Through these mechanisms, multicellular organisms are able to “sense” both their internal and external environment. Accordingly, the control of the excitability of sensory pathways is critical: a fine balance must be maintained to allow for effective detection of the environment without aberrant or excessive electrical signaling that could lead to dysfunction (excessive reflexes, pain).

Divalent cation (Ca^2+^, Mg^2+^) interaction is known to modulate the function of both soluble and structural proteins, including enzymes, membrane receptors, ion channels and transporters. Extracellular divalent cations have been previously shown to modulate excitability in neurons and other excitable cells. Extracellular divalent cations contribute substantially to the surface potential of the plasma membrane, effectively modulating the gating of voltage-gated ion channels (e.g. Na_V_, K_V_ and Ca_V_). Increasing divalent concentrations increases this charge screening effect, which results in slower gating kinetics and rightward shifts in voltage-dependent activation curves [2], [3]. Divalent cations also directly affect specific ion channels by blocking the peptide pore: for example divalents reduce the conductance of Na_V_ [4] and Ca_V_ [5] and block inward currents through TRPM7 (transient receptor potential melastatin related 7) [6] and cyclic nucleotide-gated channels [7], Ca^2+^ blocks acid-sensing ion channels [8] and Mg^2+^ blocks NMDA channels [9] and the epithelial Ca^2+^ channel [10]. In addition, divalent cations have been shown to modulate hERG K^+^ channels [11], CLC-type Cl^−^ channels [12], P2X7 channels [13] and nicotinic ACh receptors allosterically [14].

Extracellular divalent concentrations are not fixed (particularly at mucosal-air interfaces), and are subject to change due to disease and diet. The effect of divalent concentration variations on sensory nerve excitability is unclear. Studies suggest that extracellular Ca^2+^ can have conflicting effects on vagal sensory neuronal excitability due to modulation of intracellular sites (secondarily to Ca^2+^ influx) on K^+^ and Cl^−^ channels [15], [16], [17], [18]. Intriguingly, a study of airway vagal afferent nerve terminals demonstrated robust action potential discharge in response to the replacement of external Ca^2+^ with Mg^2+^ [19]. Electrophysiological evidence suggested that this response was not inhibited by blockers of K_V_ or Ca_V_. The finding that reductions in external Ca^2+^ activates vagal afferents is surprising as it is fairly common practice in Ca^2+^ imaging studies of dissociated neurons to use Ca^2+^-free external solutions (chelated with EGTA) to determine the extent to which Ca^2+^ influx or Ca^2+^ intracellular stores contribute to an increase in [Ca^2+^]_i_ during neuronal activation. In these imaging studies EGTA (Ca^2+^ depletion) does not produce any overt neuronal activation, which is contradictory to the Undem et al study [19]. We noted in preliminary studies, however, that use of EDTA did elicit robust response in sensory neurons. EDTA chiefly differs from EGTA in that it is also a high affinity Mg^2+^ chelator. In the present study, we sought to clarify the Ca^2+^/divalent sensing of vagal sensory neurons and evaluated the effect of Ca^2+^ and Mg^2+^ on dissociated mouse vagal neurons using live cell imaging and electrophysiology. The data indicate the presence of a high affinity divalent sensor on vagal neurons that is coupled to a novel voltage-gated non-selective cation current.

## Materials and Methods

All experiments were approved by the University of South Florida Institutional Animal Care and Use Committee. In total 120 mice and 2 guinea pigs were used in this study.

### Dissociation of mouse sensory ganglia

Mouse sensory ganglia (vagal, trigeminal and dorsal root ganglia) were isolated and enzymatically dissociated from wild-type C57BL/6J mice using previously described methods [20]. Isolated neurons were plated onto poly-D-lysine-coated and laminin-coated cover slips and used within 24 hours.

### HEK293 cell culture

Wild-type HEK293 cells were obtained from the sources described by Taylor-Clark et al [20]. The HEK cells were cultured as previously described [20]. Cells were maintained in an incubator (37°C, 5% CO_2_) in DMEM (containing 110 mg/l pyruvate and 564 mg/l L-Glutamine) supplemented with 10% FBS and 0.5% penicillin/streptomycin. Cells were removed from their culture flasks by treatment with Accutase (Sigma), then plated onto poly-D-lysine-coated cover slips and incubated at 37°C for >1 h before experimentation.

*Fluorescent imaging:* Cells were studied for changes in [Ca^2+^]_i_ with Fura 2AM (TEFlabs, Austin, TX, US) or [Na^+^]_i_ with SBFI (TEFlabs, Austin, TX, US). Neuron-covered coverslips were incubated (at 37°C) with Fura-2 AM (8 µM, for 40 min) or SBFI (15 µM, for 2 hours) in L-15 media containing 10% FBS. HEK293-covered coverslips were incubated (at 37°C) with SBFI (15 µM, for 2 hours) in DMEM (containing 110 mg/L pyruvate and 564 mg/L L-Glutamine) supplemented with 10% FBS. For imaging, the coverslip was placed in a custom-built heated chamber (bath volume of 300 µL) and superfused by gravity at 6 ml/min with Locke solution (35°C; composition (mM): 136 NaCl, 5.6 KCl, 1.2 MgCl_2_, 2.2 CaCl_2_, 1.2 NaH_2_PO_4_, 14.3 NaHCO3, 10 dextrose (gassed with 95% O_2_–5% CO_2_, pH 7.3–7.4)) for 15 minutes before and throughout each experiment. Changes in [Ca^2+^]_i_ (Fura 2AM) or [Na^+^]_i_ (SBFI) were monitored by sequential dual excitation, 340 and 380 nm (emission 510 nm), measured by digital microscopy (CoolSnap HQ2; Photometrics, Surrey, BC, Canada) and analyzed by specialized software (Nikon Elements; Nikon, Melville, NY, USA). The ratio images were acquired every 6 or 12 seconds. At the end of the dissociated neuronal studies, neurons were exposed to KCl (30 seconds, 75 mM) to confirm voltage sensitivity. At the end of all Fura 2AM experiments, cells were exposed to ionomycin (60 seconds, 5 µM) to obtain a maximal response. At the end of all SBFI experiments, cells were exposed to gramicidin (60 seconds, 5 µM) to obtain a maximal response. In those studies in which external Na^+^ was completely replaced by N-methyl-D-glucamine (NMDG^+^), the cells were superfused with (in mM) 154 NMDGCl, 4.7 KCl, 1.2 MgCl_2_, 2.2 CaCl_2_, 10 HEPES, 5.6 dextrose (pH (7.4) with 1.5 M NMDG). External NMDG^+^ was then replaced with equimolar Na^+^ prior to gramicidin positive control.

For the analysis of [Ca^2+^]_i_ or [Na^+^]_i_, we used the excitation ratio 340/380 (so as to avoid the necessity of calibrating the ratiometric responses to [Ca^2+^]_i_ or [Na^+^]_i_ for each cell) and relate all measurements to the peak positive response in each viable cell. Only cells that had a robust response to the positive control were included in analyses. At each time point for each cell, data was presented as the percentage change in 340/380 ratio (R), normalized to maximum response (ionomycin or gramicidin for [Ca^2+^]_i_ or [Na^+^]_i_ studies, respectively): response_x_ = 100×(R_x_−R_bl_)/(R_max_−R_bl_), where R_x_ was the 340/380 ratio of the cell at a given time point, R_bl_ was the cell's mean baseline 340/380 ratio measured over 120 s, and R_max_ was the cell's peak 340/380 ratio. All data is presented as the normalized mean ± S.E.M of all the neurons: as a % of ionomycin or gramicidin for [Ca^2+^]_i_ or [Na^+^]_i_ studies, respectively. External divalent cations were chelated by a 3 minute treatment of 5 mM EDTA (with 0 mM Mg^2+^ and 0 mM Ca^2+^ added). External Ca^2+^ was chelated by a 3 minute treatment of 5 mM EGTA (with 1.2 mM Mg^2+^ and 0 mM Ca^2+^ added).

### Patch-clamp electrophysiology

Recordings from HEK293 and vagal neurons were made using whole-cell or perforated patch-clamp techniques as indicated. Perforation was achieved using 5 µg/ml gramicidin. Patch pipettes were fabricated from 1.5 mm o.d. borosilicate glass (Sutter Instrument Co.) and fire-polished. Pipettes for perforated patch recording (1–3 MΩ) were filled with solution composed of (mM): 140 KCl, 1 CaCl_2_, 2 MgCl_2_, 10 HEPES, 10 dextrose, 11 EGTA, adjusted to pH 7.2 with NaOH. Cells on a coverslip were superfused at 8 mL/min with HEPES-buffered bath solution (34°C; composition (mM): 140 NaCl, 4.7 KCl, 1.2 MgCl_2_, 2.5 CaCl_2_, 10 HEPES, 10 dextrose, 5 tetraethylammonium (TEA), 1 4-aminopyridine (4-AP) adjusted to pH 7.4 with NaOH). Whole-cell patch-clamp recordings were made with pipettes (3–5 MΩ) filled with solution composed of (mM): 5 NaCl, 104 CsCl, 1 CaCl_2_, 2 MgCl_2_, 10 HEPES, 10 dextrose, 11 EGTA (to give 60 nM [Ca^2+^]_free_), 35 TEA and 1 4-AP, adjusted to pH 7.2 with NaOH. Cells were superfused with HEPES-buffered bath solution (34°C; composition (mM): 140 NaCl, 4.7 CsCl, 1.2 MgCl_2_, 2.5 CaCl_2_, 10 HEPES, 10 dextrose, 5 TEA, 1 4-AP adjusted to pH 7.4 with NaOH). For experiments in low Na^+^, 108 mM mannitol was substituted for 54 mM NaCl in the bath solution.

Membrane currents were recorded using a MultiClamp 700B amplifier, Digidata 1440A and pClamp 10 acquisition software (Molecular Devices). Current signals were sampled at 100 KHz and filtered at 5 KHz. Cells were routinely voltage-clamped at −120 mV and a series of depolarizing voltage steps was applied in 10 mV increments from −120 to +60 mV. Currents evoked by voltage steps were analyzed at two different time points: the peak Na_V_-like current (*I_peak_*) was measured as the peak current observed in the first 6 ms of the voltage step, and the ‘persistent’ current (*I_persistent_*) measured at 24.5 ms from the start of the step. For experiments examining the time-dependent inactivation of *I_DF_*, cells were held at −120 mV and stepped to −20 mV for 25, 525, 1025, 1525 and 2025 ms. In order to minimize the effect of Na_V_ contribution to the overall current, a number of experiments were also performed by holding cells at −120 mV, applying a 200 ms long step of −20 mV and stepping in 20 mV increments from −120 to +40 mV. Tail currents were fitted using the following equation: 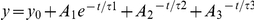. Current-voltage relationships were established in normal bath solution (Control), bath solution containing divalent chelators (as indicated) for 30–40 s and 2 min after washout (Recovery). Data were analyzed using paired or unpaired Student's t-tests as indicated.

### Chelating agents

External divalent cations were chelated using 5 mM EDTA. External Ca^2+^ was chelated using 5 mM EGTA. In some experiments external nanomolar and micromolar Ca^2+^ was clamped at the desired concentration using 5 mM EGTA with the added Ca^2+^ calculated by Maxchelator (http://www.stanford.edu/~cpatton/xlsconstants.htm). All solutions were made with de-ionized water, nominally free of divalent cations. All purchased salts were of the highest purity to reduce to the fullest extent any contamination with divalent cations

### Chemicals

SKF-96365 and cilnidipine were purchased from Tocris (Ellisville, MO). Fura 2AM and SBFI-AM were purchased from TEFLabs (Austin, TX). Tetrodotoxin and ω-conotoxin were purchased from Alomone (Israel). All other chemicals were purchased from Sigma-Aldrich (St. Louis, MO).

## Results

### Re-introduction of external divalent cations evokes a Ca^2+^ influx in sensory neurons

**Figure 1:**
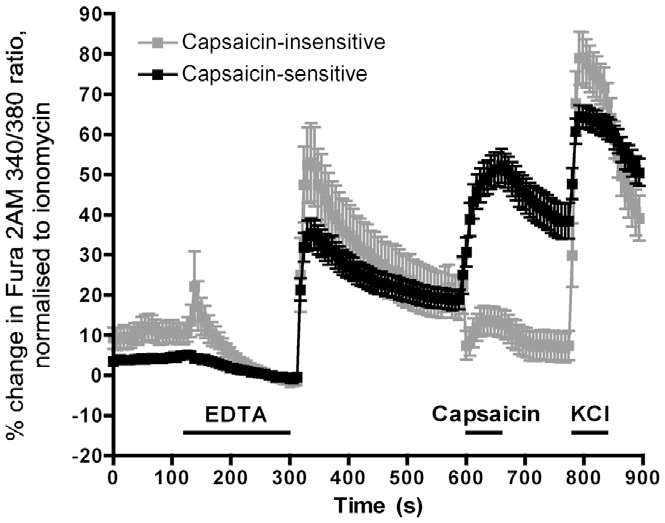
Re-introduction of divalent cations following EDTA evokes Ca^2+^ influx in vagal neurons. Mean ± S.E.M. Ca^2+^ responses of vagal neurons in response to brief treatment with EDTA (5 mM; 0 Ca^2+^, 0 Mg^2+^) followed by re-introduction of Ca^2+^ (2.2 mM) and Mg^2+^ (1.2 mM) as measured by Fura 2AM. Response to capsaicin (1 µM) and KCl (75 mM) also shown. Data comprised of capsaicin-sensitive (black squares; n = 41) and capsaicin-insensitive neurons (grey squares; n = 11) from C57BL/6 mice. Blocked line denotes application of drugs.

As part of a previous study, we had noted that the re-introduction of 2.2 mM Ca^2+^ following 10 mM EDTA treatment of guinea pig trigeminal neurons produced substantial influx of Ca^2+^, as measured by Fura 2AM [21]. We repeated these studies in dissociated mouse sensory neurons from the vagal, trigeminal and dorsal root ganglia (DRG) using ratiometric Ca^2+^ imaging (Fura 2AM). Treatment with 5 mM EDTA (0 mM Ca^2+^, 0 mM Mg^2+^) for 3 minutes led to a slow and minor decrease in [Ca^2+^]_i_. Upon re-introduction of divalents (2.2 mM Ca^2+^, 1.2 mM Mg^2+^), an increase in [Ca^2+^]_i_ was observed in 46 of 52 vagal neurons. The neurons were further characterized by their response to capsaicin (a selective transient receptor potential vanilloid receptor 1 (TRPV1) agonist) – a hallmark of nociceptive C-fiber nerves involved in the detection of noxious stimuli [22]. Both capsaicin-sensitive (likely nociceptive) and capsaicin-insensitive (likely non-nociceptive) neurons responded to the divalent re-introduction, with a mean response of 35.3±3.8% and 52.3±9.4% of ionomycin, respectively (Fig. 1). Repeated withdrawal and re-introduction of divalents caused reproducible responses (data not shown). Similar responses to divalent re-introduction following treatment with EDTA (0 mM Ca^2+^, 0 mM Mg^2+^) were observed in trigeminal neurons (mean response of 30.5±3.3% and 34.3±3.8% of ionomycin, for capsaicin-sensitive and capsaicin-insensitive neurons respectively) and DRG neurons (mean response of 35.4±3.3% and 45.0±3.7% of ionomycin, for capsaicin-sensitive and capsaicin-insensitive neurons respectively) (Table 1 and data not shown). These data demonstrate that brief withdrawal of Ca^2+^ and Mg^2+^ from mouse sensory neurons activates a mechanism that upon re-introduction of these divalents induces an increase in [Ca^2+^]_i_. Such responses are reminiscent of Ca^2+^ ‘addback’ responses following Ca^2+^ store-depletion in non-neuronal cells through Ca^2+^-permeable store-operated channels (SOC) [23].

**Table 1:** Re-introduction of divalent cations following EDTA activates mouse sensory neurons.

	Vagal	DRG	Trigeminal
Capsaicin-sensitive	36/41	34/38	23/25
Capsaicin-insensitive	10/11	46/51	37/41

Data represent the number of sensory neurons (of the total tested) activated by re-introduction of divalent cations following brief treatment with EDTA (5 mM; 0 Ca^2+^, 0 Mg^2+^) as measured by Fura 2AM. Neurons were characterized by their positive response to capsaicin (1 µM). All neurons responded to KCl (75 mM).

**Figure 2:**
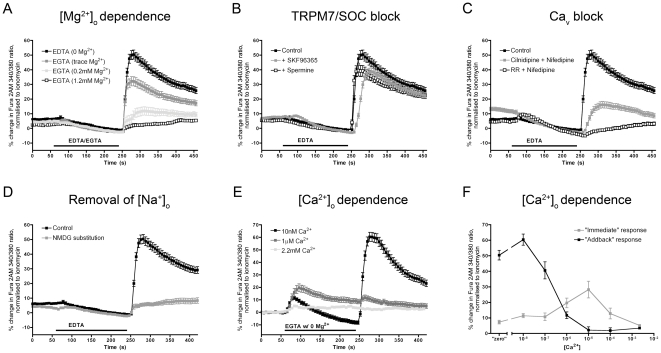
Pharmacological analysis of Ca^2+^ influx in vagal neurons in response to divalent cation removal/re-introduction. Mean ± S.E.M. Ca^2+^ responses of vagal neurons as measured by Fura 2AM. Blocked line denotes the chelation of divalent cations, at all other times Ca^2+^ and Mg^2+^ are at 2.2 mM and 1.2 mM, respectively. All neurons responded to KCl (75 mM). A, the response to divalent chelation (5 mM EDTA, 0 Ca^2+^, 0 Mg^2+^) or Ca^2+^ chelation (5 mM EGTA, 0 Ca^2+^) in combination with various Mg^2+^ concentrations (0, 0.2 and 1.2 mM). B, the effect of SOC blocker SKF96365 (10 µM; grey squares) or TRPM7 blocker spermine (20 µM; black outlined boxes) on the response to EDTA (5 mM; 0 Ca^2+^, 0 Mg^2+^). C, the effect of a combination of clinidipine and nifedipine (both 10 µM; grey squares) or a combination of ruthenium red (RR, 30 µM) and nifedipine (10 µM; black outlined boxes) on the response to EDTA (5 mM; 0 Ca^2+^, 0 Mg^2+^). D, the effect of total external Na^+^ replacement with NMDG^+^ (grey squares) on the response to EDTA (5 mM; 0 Ca^2+^, 0 Mg^2+^). E and F, the response to the absence of Mg^2+^ combined with various Ca^2+^ concentrations titrated with 5 mM EGTA (resulting in 0 nM (n = 94), 10 nM (n = 56), 100 nM (n = 32), 1 µM, (n = 63), 10 µM, (n = 47), 100 µM, (n = 58), 2.2 mM (n = 21)). E, Mean ± S.E.M. Ca^2+^ responses against time demonstrating the ‘immediate’ Ca^2+^ response observed *during* treatment with low divalent cations and the ‘addback’ response observed following re-introduction of divalent cations (Ca^2+^ 2.2 mM, Mg^2+^ 1.2 mM). F, maximal response observed during ‘immediate’ response (grey squares) and during ‘addback’ response (black squares).

We investigated the role of Mg^2+^ in the responses of vagal neurons to external divalent cation withdrawal. EDTA chelates all divalent (and trivalent) cations with high affinity, whereas EGTA chelates all divalent (and trivalent) cations except Mg^2+^ with high affinity [24]. As such we were able to fully chelate Ca^2+^ with EGTA and substitute varying [Mg^2+^]. Upon re-introduction of divalents (2.2 mM Ca^2+^, 1.2 mM Mg^2+^) following complete divalent chelation with EDTA, vagal neurons responded with a mean increase in [Ca^2+^]_i_ of 50.4±3.1% of ionomycin (n = 94). In contrast, vagal neurons failed to responded to re-introduction of 2.2 mM Ca^2+^ following Ca^2+^ chelation with EGTA (n = 89; Fig. 2A). Lowering the [Mg^2+^] in the EGTA solution from 1.2 mM to 0.2 mM (n = 46) and 0 mM (trace, n = 71) resulted in a progressive increase in [Ca^2+^]_i_ following re-introduction of divalents, indicating that withdrawal of Mg^2+^ is critical to these responses. To test whether or not withdrawal and re-introduction of Mg^2+^ is sufficient to elicit an increase in [Ca^2+^]_i_, we compared responses of vagal neurons to 3 min treatment of 0 mM Mg^2+^ (trace), with and without chelation of Ca^2+^ with EGTA. Withdrawal and re-introduction of Mg^2+^ alone had no significant effect on [Ca^2+^]_i_ (n = 21), indicating that withdrawal of Ca^2+^ is also critical to these responses (see below and Fig. 2E and F).

Based on these data, we initially identified SOC and TRPM7 as candidate molecular correlates underlying the mechanism for the increase in [Ca^2+^]_i_ following divalent re-introduction. SOC (e.g. Orai 1) are Ca^2+^ permeable plasma membrane ion channels that are typically activated following depletion of Ca^2+^ stores in the endoplasmic reticulum through the actions of Ca^2+^ sensor proteins (STIM) [25]. TRPM7 are ubiquitously expressed non-selective cation channels that can be gated by decrements in extracellular [Ca^2+^] and [Mg^2+^] [26]. To test these hypotheses, we added either 10 µM SKF96365 (SOC blocker [23]) or 20 µM spermine (TRPM7 blocker [27]) to the external solutions. Block of SOC (n = 112) or TRPM7 (n = 59) failed to inhibit the increase in [Ca^2+^]_i_ during re-introduction of divalents (2.2 mM Ca^2+^, 1.2 mM Mg^2+^) following treatment with EDTA (0 mM Ca^2+^, 0 mM Mg^2+^; Fig. 2B).

### Na^+^ influx is required for the Ca_V_-mediated Ca^2+^ influx upon re-introduction of divalent cations

Vagal sensory neurons contain many Ca^2+^ permeable channels including TRPV1, TRPM8, TRPA1 and N-type, L-type and T-type voltage-gated Ca^2+^ channels. It is unlikely that TRPV1, TRPM8, TRPA1 are responsible for our observed responses to external divalent modulation as only nociceptive subsets of vagal neurons express these channels [28] but the divalent response was universal. We tested the contribution of voltage-gated Ca^2+^ channels to the increase in [Ca^2^]_i_ using two separate Ca_V_ inhibitor solutions. A combination of 10 µM nifedipine (L-type blocker) and 10 µM cilnidipine (N-type and L-type blocker) significantly reduced the response of vagal neurons to the re-introduction of divalents (2.2 mM Ca^2+^, 1.2 mM Mg^2+^) following treatment with EDTA (0 mM Ca^2+^, 0 mM Mg^2+^) from 50.4±3.1% (n = 94) to 16.2±2.2% of ionomycin (n = 37; p<0.0001) (Fig. 2C). Similarly a combination of 10 µM nifedipine and 30 µM ruthenium red (non-selective Ca_V_ blocker [29]) abolished the response of vagal neurons to the re-introduction of divalents (n = 93). However, when we completely replaced the external Na^+^ with an equimolar concentration of the large, generally channel-impermeable, NMDG^+^ the response of vagal neurons to the re-introduction of divalents following treatment with EDTA was completely abolished (n = 45; Fig. 2D). Taken together these results suggest that the influx of Ca^2+^ (following divalent re-introduction) through Ca_V_ is first dependent on the influx of Na^+^. It is likely that the increase in [Ca^2+^]_i_ observed is a consequence of neuronal depolarization (Na^+^ influx) occurring during the withdrawal of external divalent cations (EDTA treatment).

Lastly, we evaluated the sensitivity of these responses to external Ca^2+^. Vagal neurons were treated with Mg^2+^-free buffer containing various Ca^2+^ concentrations (titrated by EGTA chelation). Treatment of vagal neurons with sub-millimolar Ca^2+^ (and zero Mg^2+^) evoked two distinct responses (Fig. 2E and F). At very low Ca^2+^ concentrations (10 nM and 100 nM), Ca^2+^ influxes occurred upon re-introduction of divalents, similar to EDTA-induced responses. Increasing the external Ca^2+^ concentration reduced this ‘addback’ response. However, treatment with low micromolar Ca^2+^ (and zero Mg^2+^) evoked an immediate Ca^2+^ influx that was not significantly increased following the re-introduction of millimolar Ca^2+^ (suggesting that the Ca_V_ are not activated during the re-introduction). Further increasing the Ca^2+^ concentration reduced this ‘immediate’ response so that treatment with 2.2 mM Ca^2+^ (and zero Mg^2+^) failed to evoke either responses (Fig. 2E and F). The data suggests that in the absence of external Mg^2+^, these vagal neurons are activated by external [Ca^2+^]<100 µM. With nanomolar external [Ca^2+^], the prevailing response is a Na^+^-dependent depolarization-induced Ca^2+^ influx through Ca_V_ following divalent re-introduction. With micromolar external [Ca^2+^], the putative channel is preferentially permeable to Ca^2+^, thus an immediate Ca^2+^ influx is observed and the Na^+^-dependent depolarization-induced Ca^2+^ influx through Ca_V_ following divalent re-introduction is absent.

### Withdrawal of external divalent cations evokes a Na^+^ influx in sensory neurons that is not inhibited by blockers of Na_V_ or Ca_V_

**Figure 3:**
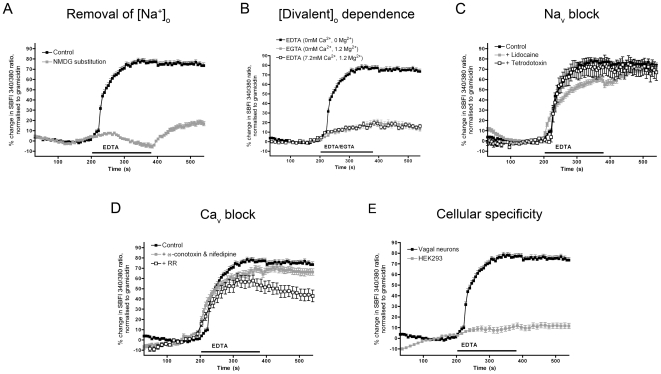
Chelation of external divalent cations evokes Na^+^ influx in sensory neurons. Mean ± S.E.M. Na^+^ responses of vagal neurons as measured by SBFI. Blocked line denotes the chelation of divalent cations, at all other times Ca^2+^ and Mg^2+^ are at 2.2 mM and 1.2 mM, respectively. A, the response to divalent chelation (5 mM EDTA; 0 Ca^2+^, 0 Mg^2+^) in the presence of external Na^+^ (black squares) or external NMDG^+^ (grey squares). B, the response to chelation of Ca^2+^ alone (5 mM EGTA; 0 Ca^2+^, 1.2 mM Mg^2+^; grey squares) or the response to 5 mM EDTA saturated with 7.2 mM Ca^2+^ and 1.2 mM Mg^2+^ (black outlined boxes). C, the effect of lidocaine (1 mM; grey squares) or TTX (1 µM; black outlined boxes) on the response to 5 mM EDTA (0 Ca^2+^, 0 Mg^2+^). D, the effect of a combination of ω-conotoxin (1 µM) and nifedipine (10 µM; grey squares) or ruthenium red (RR, 30 µM, black outlined boxes) on the response to 5 mM EDTA (0 Ca^2+^, 0 Mg^2+^). E, the response to 5 mM EDTA (0 Ca^2+^, 0 Mg^2+^) in vagal neurons (black squares) and HEK293 cells (grey squares).

Using ratiometric Na^+^ imaging (SBFI) of dissociated mouse vagal sensory neurons, we found that treatment with EDTA (0 mM Ca^2+^, 0 mM Mg^2+^) induced an increase in [Na^+^]_i_ in 92 of 106 neurons: mean response 78.4±2.5% of gramicidin (Fig. 3A). To confirm the SBFI imaging was dependent on Na^+^ influx, the external Na^+^ was replaced with equimolar NMDG^+^ and the observed EDTA response was abolished (n = 33; Fig. 3A). As with the Fura 2AM Ca^2+^ studies, chelating only Ca^2+^ using EGTA in the presence of Mg^2+^ failed to significantly activate vagal neurons (n = 39), indicating that Na^+^ influx occurred as a result of both Ca^2+^ and Mg^2+^ depletion (Fig. 3B). In addition, treatment with 5 mM EDTA saturated with divalents (7.2 mM Ca^2+^, 1.2 mM Mg^2+^) also failed to activate vagal neurons (n = 25), indicating that neuronal activation was specifically dependent on divalent chelation rather through some other action of EDTA itself.

We next evaluated the contribution of voltage-gated channels in the Na^+^ influx following external divalent depletion. Vagal sensory neurons express a number of Na_V_, including tetrodotoxin (TTX)-sensitive Na_V_1.7 and the TTX-resistant Na_V_1.8 and Na_V_1.9 [30]. Pretreatment of the neurons with 1 µM TTX had no effect on EDTA-induced responses (n = 41; Fig. 3C). Pretreatment with 1 mM lidocaine, a concentration that inhibits both TTX-sensitive and TTX-resistant neuronal Na_V_ currents [31], also failed to inhibit the EDTA-induced Na^+^ influx (n = 39).

Previous studies including those of chick DRG neurons have demonstrated that Ca_V_ are able to conduct Na^+^ ions in the absence of external Ca^2+^ [5]. Given that the Ca^2+^ influx following re-introduction of divalents was reduced by Ca_V_ inhibition, we tested the role of these channels in the Na^+^ influx. Pretreatment of the neurons with a combination of 1 µM ω-conotoxin (potent N-type channel toxin) and 10 µM nifedipine no significant effect on EDTA-induced responses (n = 45; Fig. 3D). In addition, pretreatment with 30 µM ruthenium red, a blocker of Ca_V_ [29], also failed to overtly inhibit the EDTA-induced Na^+^ influx (n = 16).

The Ca^2+^ and Na^+^ imaging studies of dissociated mouse neurons indicate that chelation of both Ca^2+^ and Mg^2+^ is required for the influx of Na^+^ which (likely through depolarization) leads to the activation of Ca_V_ that then allows for Ca^2+^ influx upon re-introduction of external Ca^2+^. Inhibition studies suggest that the Na^+^ influx in response to external divalent chelation is not via SOC, TRPM7, Na_V_ or Ca_V_ channels. In order to evaluate whether or not the EDTA-induced Na^+^ influx was a non-specific effect on membrane integrity we compared the responses of mouse vagal neurons with HEK293 cells in our SBFI assay. Unlike vagal neurons, HEK293 failed to demonstrate Na^+^ influx in response to a treatment with EDTA (0 mM Ca^2+^, 0 mM Mg^2+^; n = 36; Fig. 3E). This suggests that the Na^+^ influx is due to the expression of specific divalent-sensitive molecular identities that are present in mouse sensory neurons but not in HEK293 cells.

### Withdrawal of external divalent cations evokes a voltage-gated current (I_DF_) in sensory neurons

**Figure 4:**
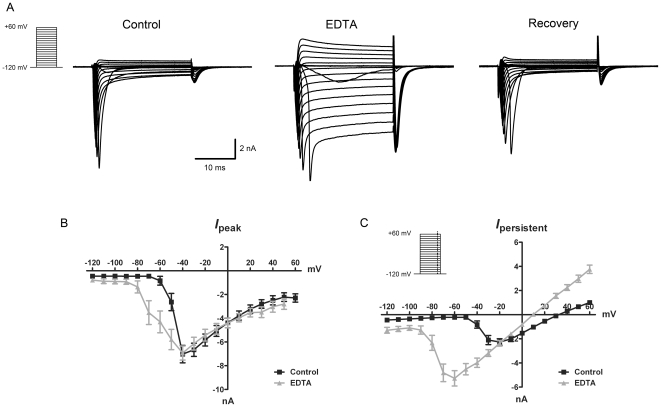
Chelation of external divalent cations activates a voltage-gated current in vagal neurons. Mean ± S.E.M. A, voltage steps of 10 mV increments were applied to vagal neurons held at −120 mV. Neurons were perfused with normal bath solution (Control and Recovery) or bath solution containing 5 mM EDTA with nominally 0 mM Ca^2+^ and 0 mM Mg^2+^ (EDTA). The resulting currents are depicted. B, the current-voltage relationship of the peak amplitude of the fast component of the current (n = 8 and 14 for Control (black squares) and EDTA (grey triangles) respectively). C, the current-voltage relationship of the persistent component of the current recorded at 24.5 ms after the onset of the voltage step (indicated by dotted line in inset; n = 24).

To further characterize the response of mouse vagal neurons to chelation of external divalent cations we used whole-cell patch-clamp electrophysiology. In order to eliminate K^+^ currents, we replaced K^+^ with equimolar Cs^+^ and added TEA and 4-AP to both external and pipette solutions (see methods for details). Vagal neurons were held at −120 mV and subjected to 25 ms depolarizing steps from −120 mV to +60 mV every 10 or 20 seconds. In control conditions (2.5 mM Ca^2+^, 1.2 mM Mg^2+^), these pulses evoked a complex current: an Na_V_-like fast multi-component inward current with fast inactivation and an extrapolated reversal potential (+90 mV) consistent with Na^+^ selectivity – likely due to the activation of Na_V_1.7 and 1.8; and a uncharacterized smaller slowly inactivating persistent current with a reversal potential at approximately +30 mV. These currents are consistent with other studies of sensory neurons [30], [32]. 40 sec treatment with 5 mM EDTA (0 mM Ca^2+^, 0 mM Mg^2+^) caused dramatic changes in the current profile until the EDTA was washed away and the divalents were re-introduced, at which point the currents returned to baseline (Fig. 4A). The Na_V_-like currents were modulated by external divalent withdrawal (Fig. 4B): peak activation was shifted to the left (n = 18; p<0.005) and the activation was faster (time to peak when stepped to −50 mV was 2.6±0.2 ms and 1.5±0.1 ms in control and EDTA-treatment, respectively (n = 8 and 14 respectively; p<0.005)), whereas peak amplitude was unchanged – as would be predicted for the removal of divalent cation charge screening of the membrane [2], [3]. However, chelation of external divalent cations (*divalent-free*) also evoked a large voltage-gated ‘persistent’ current in all neurons tested (*I_DF_*; n = 24; Fig. 4A and C). The *I_DF_* was absent at very negative potentials but was typically activated by depolarization to steps of −70 mV and above and reversed at approximately +10 mV. The magnitude of tail currents after the 25 ms step were not appreciably different for steps above −60 mV indicating complete activation of the unknown channels at these voltages (e.g. −10.5 ±1.1 nA and −10.5 ±0.9 nA for steps to −50 mV and +60 mV, respectively). The *I_DF_* was apparent either when using gramicidin (5 µg/ml) to obtain perforated patches (n = 7; data not shown) or in whole-cell patch-clamp recordings (n = 24). We briefly repeated these studies in guinea pig vagal neurons using whole-cell patch-clamp recording and found a similar voltage-gated persistent *I_DF_* (n = 5; data not shown). Finally, to determine if the evoked *I_DF_* was a non-specific effect on membrane integrity or patch pipette seal resistance, we performed whole-cell patch-clamp electrophysiology on HEK293 cells. 5 mM EDTA treatment failed to evoke a significant voltage-activated *I_DF_* current in HEK293 cells (n = 5, data not shown), indicating that the *I_DF_* observed in sensory neurons is likely due to the selective expression of specific divalent-sensitive molecular identities.

**Figure 5:**
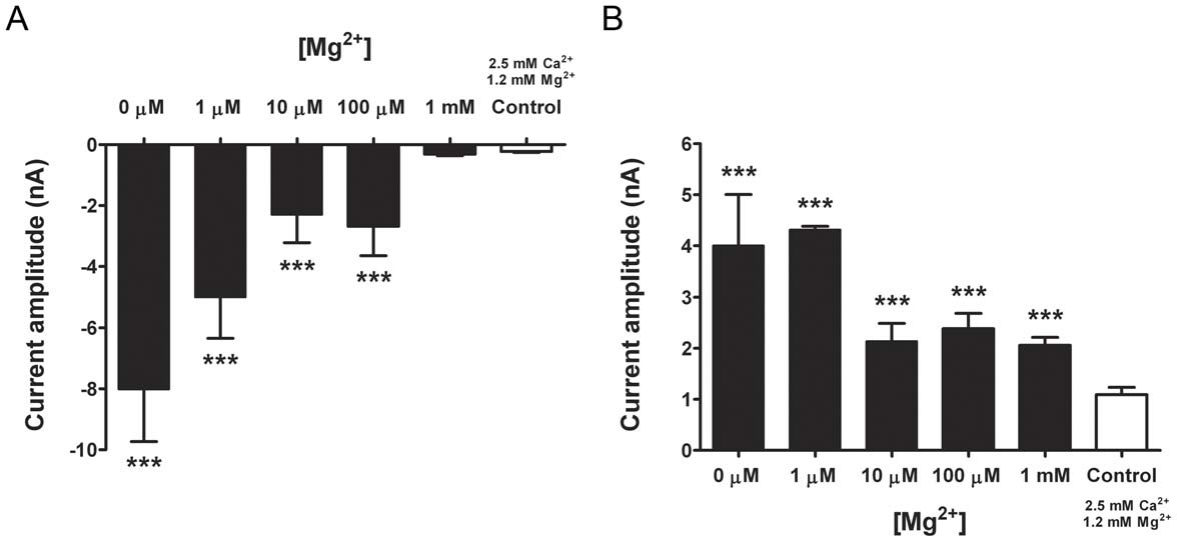
Increasing external [Mg^2+^] inhibits *I_DF_* activation. Mean ± S.E.M. Vagal neurons were held at −120 mV and stepped to −70 mV to record the inward current (A) or stepped to +60 mV to record the resulting outward current (B). Neurons were perfused with normal bath solution containing 2.5 mM Ca^2+^ and 1.2 mM Mg^2+^ (Control; white bar), or solution containing 5 mM EGTA and different [Mg^2+^] (0 µM, 1 µM, 10 µM, 100 µM and 1 mM; solid black bars). Statistical analysis was carried out between control conditions and increasing [Mg^2+^] using the unpaired Student's t-test (*** p<0.005).

We next evaluated the role of Ca^2+^ and Mg^2+^ in the evoked *I_DF_* in vagal neurons. As with the SBFI Na^+^ imaging data, treatment with 5 mM EDTA saturated with divalents (7.2 mM Ca^2+^, 1.2 mM Mg^2+^) failed to activate the neurons (n = 2; data not shown). Consistent with a requirement for both Ca^2+^ and Mg^2+^ depletion, EGTA (0 mM Ca^2+^, 1 mM Mg^2+^) also failed to evoke a significant *I_DF_* (n = 3) – although we nonetheless observed a shift in the Na_V_-like current profiles consistent with a reduction in charge screening (e.g. time to peak was faster (n = 3; p<0.01; data not shown)). Decreasing the [Mg^2+^] in the Ca^2+^-free EGTA external solution evoked currents similar to EDTA (Fig. 5A and B). It should be noted that the time required to evoke a response with solutions containing 100 µM Mg^2+^ was on occasion increased from 30 to 60 s (3/6 cells). Consistent with our Fura 2AM Ca^2+^ imaging data, where external [Mg^2+^] greater than 200 µM effectively inhibited Ca^2+^ influx, 100 µM Mg^2+^ (and 0 mM Ca^2+^) produced noticeably smaller *I_DF_* currents compared to cells treated with EDTA (0 mM Ca^2+^, 0 mM Mg^2+^). There was little difference between the Mg^2+^ inhibition of *I_DF_* at −70 mV and +60 mV (IC_50_ of 1.2 µM and 3.6 µM, respectively), which suggests that Mg^2+^ external pore block is not the predominant mechanism by which Mg^2+^ inhibits *I_DF_*.

**Figure 6:**
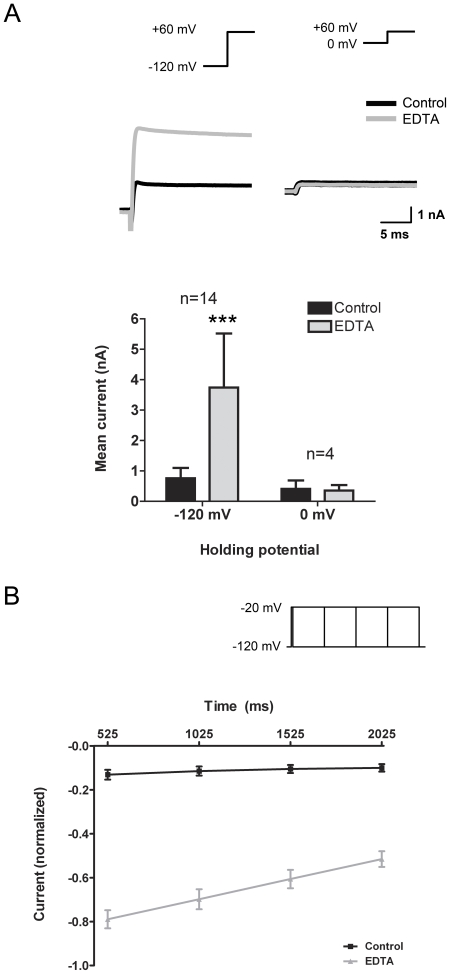
*I_DF_* exhibits voltage- and time-dependent inactivation. A, vagal neurons were held at either −120 mV or 0 mV and stepped to +60 mV for 25 ms. Current recordings are shown for a typical neuron in control solution and 5 mM EDTA (0 Ca^2+^, 0 Mg^2+^), and mean ± S.E.M. data are shown below. Statistical analysis was carried out between control conditions and addition of 5 mM EDTA using the paired Student's t-test (*** p<0.005). B, vagal neurons held at −120 mV were stepped to −20 mV for different durations: 25, 525, 1025, 1525 and 2025 ms. The peak amplitude of the tail currents (mean ± S.E.M) for the 525, 1025, 1525 and 2025 ms time points was normalized to the current recorded at the 25 ms (n = 8).

The inactivation characteristics of *I_DF_* were briefly studied. The voltage-dependence of *I_DF_* was tested by holding vagal neurons at either −120 mV or 0 mV, then stepping to +60 mV for 25 ms. We studied the evoked outward current in control conditions and with EDTA (0 mM Ca^2+^, 0 mM Mg^2+^) treatment. Chelation of external divalents evoked a robust outward current in steps from −120 mV but not in steps from 0 mV (Fig. 6A). These results indicate that *I_DF_* had undergone voltage-dependent inactivation. The rate of inactivation was examined by subjecting cells to a series of depolarizing steps to −20 mV (from −120 mV) of increasing duration (25 ms to 2025 ms) and, following the return to −120 mV, inward tail currents were recorded. Under control conditions tail current amplitude decreased over time to a small plateau consistent with the presence of Na_V_1.8 [30]. During treatment with EDTA (0 mM Ca^2+^, 0 mM Mg^2+^) tail current amplitude from the evoked *I_DF_* decreased over time (n = 8; p<0.0001), indicating that the *I_DF_* was indeed slowly inactivating (Fig. 6B). Upon repolarization to −120 mV, the tail currents deactivated with two exponential functions: 0.18±0.03 and 0.89±0.14 ms for the fast and slower time constants, respectively (data not shown). In separate experiments, *I_DF_* deactivation was found to display voltage dependence, occurring consistently at membrane potentials more negative than −60 mV (data not shown).

### I_DF_ is a non-selective cation current that is not inhibited by blockers of Na_V_, Ca_V_ or TRPM7 channels

**Figure 7:**
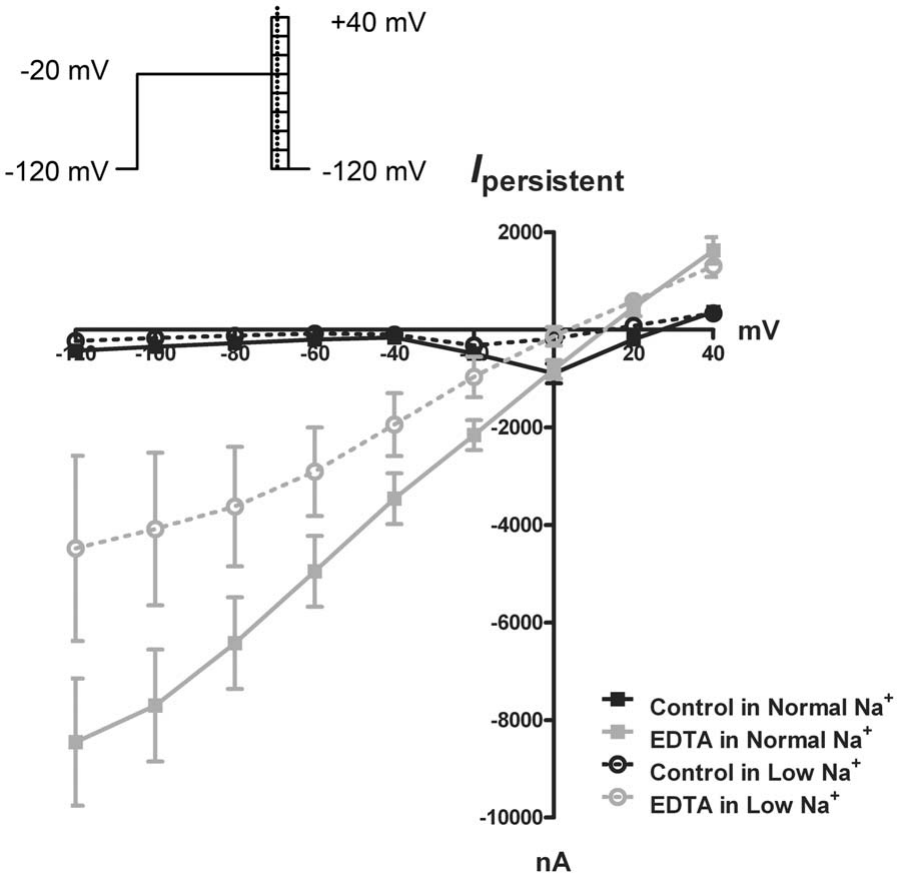
The *I_DF_* is a Na^+^-permeable non-selective cation current. Mean ± S.E.M. Vagal neurons were held at −120 mV, prepulsed to −20 mV for 200 ms and then stepped from −120 to +40 mV in 20 mV increments for 25 ms (current measured at 1 ms). The current-voltage relationship from this Na_V_ inactivation protocol is shown for cells bathed in “Normal Na^+^” 154 mM NaCl-containing solutions: control external solution (solid line, black squares) and solution containing 5 mM EDTA (0 Ca^2+^, 0 Mg^2+^) (solid line, grey squares; n = 15). Also depicted are the current-voltage relationships for cells bathed in “low” 100 mM NaCl-containing solutions: control external solution (dotted line, empty black circles) and solution containing 5 mM EDTA (0 Ca^2+^, 0 Mg^2+^; dotted line, empty grey circles; n = 6).

Mouse vagal ganglia demonstrated complex Na_V_-like currents, likely due to the expression of Na_V_1.7 and Na_V_1.8. Unlike Na_V_1.7 that can be effectively blocked by the selective toxin TTX, there are no selective and completely effective blockers of Na_V_1.8. In order to gain more information on the I–V relationship of *I_DF_* we employed a long depolarizing pulse (to −20 mV from −120 mV for 200 ms) prior to a series of voltage steps in order to reduce to the fullest extent (through inactivation) the Na_V_-like currents. These pulses should allow for the characterization of the current passed by the activated divalent-sensitive channel (with the assumption that a significant portion of the channels have not inactivated after the 200 ms depolarization). Treatment with EDTA (0 mM Ca2+, 0 mM Mg2+) evoked a current that reversed at approximately +13 mV (Fig. 7). When external [NaCl] was reduced (replaced with mannitol) to 100 mM, the *I_DF_* inward currents were reduced and the reversal potential was shifted leftward by about 10 mV, suggesting that Na+ is the major inward charge carrier with these solutions. It should be noted that a reduction in NaCl would also reduce the ionic strength of the external solution, causing a theoretical (minor) *increase* in divalent chelation by EDTA (http://www.stanford.edu/~cpatton/xlsconstants.htm).

**Figure 8:**
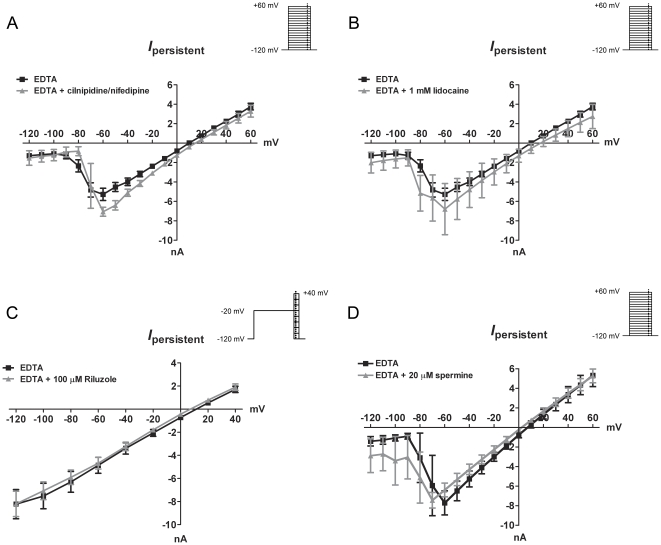
Pharmacological characterization of *I_DF_* responses in vagal neurons. Mean ± S.E.M. The current-voltage relationship of the *I_DF_* in bath solution containing 5 mM EDTA (0 Ca^2+^, 0 Mg^2+^) in the absence (black squares; n = 24) and presence of different ion channel blockers (grey triangles). A, N-type and L-type Ca_V_ blockers; 10 µM cilnidipine and 10 µM nifedipine respectively (n = 4), B, 1 mM lidocaine (n = 4), C, 100 µM riluzole (n = 4) and D, 20 µM spermine (n = 8). Insets indicate the voltage-step protocol used. A, B and C show unpaired data, D shows paired data.

Finally, the role of Ca_V_ and Na_V_ and TRPM7 in the *I_DF_* was assessed. Previous studies have demonstrated that in the absence of external Ca^2+^, Ca_V_ can conduct monovalent cations (in particular Na^+^) [5]. We compared the evoked *I_DF_* in control neurons with neurons exposed to combined treatment of 10 µM nifedipine and 10 µM cilnidipine, which substantially reduced depolarization-induced Ca^2+^ influx in Fura 2AM studies (Fig. 2C) and would be predicted to reduce L-type and N-type currents by over 70% [33],[34]. Combined inhibition of L-type and N-type failed to reduce *I_DF_* (n = 4; Fig. 8A). We also studied the effect of Na_V_ blockers of the *I_DF_*. Pretreatment of neurons with 1 mM lidocaine (5 min) caused a 65% reduction in the peak Na_V_-like current evoked by 25 ms depolarizing steps, consistent with the presence of multiple Na_V_ channels (e.g. with a step to −40 mV, the peak fast inward current was −7.0±0.7 nA and −2.4±1.9 nA in control and lidocaine treatments, respectively (p<0.005; data not shown)). However, lidocaine failed to inhibit the *I_DF_* (n = 4; Fig. 8B). To confirm a lack of contribution of Na_V_ to the *I_DF_* we used the inhibitor riluzole. Riluzole (100 µM) has been shown to inhibit numerous Na_V_ currents including ‘persistent’ currents [35]. Studies have shown riluzole block is more effective when coinciding with Na_V_ inactivation, thus we employed a 200 ms pre-pulse step from −120 mV to −20 mV prior to a series of voltages. 100 µM riluzole failed to reduce the *I_DF_* (n = 4; Fig. 8C). Lastly, having examined the lack of contribution of voltage-gated ion channels we wanted to rule out possible involvement of the TRPM7 channel that, in the absence of external Ca^2+^ and Mg^2+^, conducts monovalent cations. Using our standard voltage-step protocol (holding at −120 mV and stepping in 10 mV increments to +60 mV) vagal neurons were treated with EDTA (0 mM Ca^2+^, 0 mM Mg^2+^) until the *I_DF_* was detected, then 20 µM spermine (TRPM7 inhibitor) was added to the external solution. Consistent with the lack of effect of spermine on the EDTA-induced Ca2+ influx (Fig. 2B), spermine failed to block the *I_DF_* (n = 8; Fig. 8D).

## Discussion

This study demonstrates that a reduction of external divalent cations to micromolar concentrations activates mammalian sensory nerves via the gating of a voltage-dependent non-selective cation current. At present it is unclear if this current is produced by a single channel population or multiple channel populations, although our data describe a channel(s) that is/are activated by depolarization and undergoes time- and voltage-dependent slow inactivation. Judging from the substantial current density measured (>200 pA/pF) following current activation, we predict that the putative channel(s) underlying the *I_DF_* either (1) has/have large conductance or (2) expressed in high numbers or both.

A striking characteristic of this study is the absolute requirement for both Ca^2+^ and Mg^2+^ depletion for any neuronal activation to occur. We have shown in Ca^2+^ imaging, Na^+^ imaging and patch-clamp electrophysiology that ‘complete’ chelation of Ca^2+^ by EGTA with normal Mg^2+^ (1.2 mM) fails to evoke the current. Similarly, reduction of Mg^2+^ to trace concentrations (estimated to be <1 µM) failed to evoke significant responses. When both Ca^2+^ and Mg^2+^ were depleted, however, over 90% of neurons responded. Our data show that the divalent sensor can be almost completely inhibited by [Ca^2+^]_o_≥100 µM (IC_50_∼0.5 µM) or by [Mg^2+^]_o_≥200 µM (IC_50_∼3 µM). All our sub-millimolar Ca^2+^ concentrations were titrated with either EGTA or EDTA so it is theoretically possible that the neuronal activation was due to a depletion of other trace multivalent cations present in the solution (i.e. independent of Ca^2+^). Both EGTA and EDTA chelate multivalent cations (with the obvious exception of Mg^2+^ not being chelated by EGTA) with very high affinity (http://www.stanford.edu/~cpatton/xlsconstants.htm). However, our studies of neuronal responses to various concentrations of external Ca^2+^ (Fig. 2E and F), suggest that indeed Ca^2+^ is capable of directly affecting the divalent sensor in sensory neurons. In these experiments the fixed EGTA concentration would have maintained the multivalent cation-free environment regardless of the Ca^2+^ concentration as almost all divalent cations (e.g. Fe^2+^, Zn^2+^, Cu^2+^, Ni^2+^) have affinities for EGTA several orders of magnitude higher than Ca^2+^. Therefore the inhibition of neuronal activation must be dependent on Ca^2+^ specifically.

When the putative channel(s) is activated in the absence of external Ca^2+^ we observed Na^+^ influx, which was absent when external Na^+^ was replaced with the large cation NMDG^+^. In unclamped neurons Na^+^ influx induced the activation (likely through depolarization) of voltage-gated Ca_V_ that are expressed in all sensory neurons. Activated L-type and N-type Ca_V_ do not undergo rapid inactivation and so, upon re-introduction of Ca^2+^ into the external solutions, they were able to conduct a substantial Ca^2+^ influx (the ‘addback’ response). This response was eliminated by combinations of Ca_V_ inhibitors and also by Na^+^ replacement with NMDG^+^. Crucially, inhibition of Ca_V_ failed to prevent the Na^+^ influx, confirming that the ‘addback’ Ca^2+^ influx through Ca_V_ was triggered by Na^+^ influx through a Ca_V_-independent mechanism. Whole-cell patch-clamp studies support the hypothesis that this channel is permeable to Na^+^ ions as the inward current at negative potentials was reduced when external NaCl was partially replaced with mannitol. With our solutions the reversal potential of the *I_DF_* (near zero) suggests that the putative channel(s) is also permeable to cations other than Na^+^: outward currents were observed using either Cs^+^ (e.g. Fig. 4 and 7) or K^+^ (data not shown) in the pipette solution. In addition our Fura 2AM experiments suggest that the putative channel(s) is Ca^2+^ permeable (Fig. 2E and F).

Depletion of external divalent cations evoked a substantial voltage-gated Na^+^ current in over 95% of vagal sensory neurons. Vagal sensory neurons express voltage-gated Na_V_ 1.6, 1.7, 1.8 and 1.9 [30] and are responsible for the fast Na^+^ currents evoked by neuronal depolarization (Fig. 4A and 4B). Previous studies have shown that a reduction in divalent cations would reduce charge screening, which would result in faster gating kinetics and leftward shifts in voltage-dependent activation curves [2], [3]. We confirmed these effects. However, based on the following evidence we believe that Na_V_ are not responsible for the *I_DF_*: 1) The *I_DF_* was not inhibited by high concentrations of lidocaine or riluzole, the Na^+^ influx (SBFI recordings) was not inhibited by high concentrations of lidocaine or TTX, and the Ca^2+^ influx upon re-introduction of divalents (Fura 2AM recordings) was not inhibited by TTX (data not shown). 2) The *I_DF_* has a near zero reversal potential inconsistent with very high Na^+^ selectivity. 3) Peak Na_V_ current amplitude was unchanged by a reduction of external divalent cations. 4) The inactivation kinetics of *I_DF_* suggest very slow inactivation over seconds rather than milliseconds (typical for sensory nerve Na_V_ [30]). Indeed a reduction of charge screening as occurs with external divalent depletion would be predicted to quicken Na_V_ inactivation rather than slow it. 5) We observed charge screening effects on the Na_V_-like currents but failed to detect *I_DF_* when neurons were treated with Ca^2+^-free solutions containing 1.2 mM Mg^2+^. 6) The *I_DF_* was absent in HEK293 cells transiently transfected with human Na_V_1.7 (data not shown).

Nevertheless, the presence of Na_V_-like currents precluded precise analysis of the *I_DF_* kinetics, which likely must wait until the putative channel(s) has been cloned. Other known channels were investigated for their role as the divalent sensor in mouse vagal neurons: *I_DF_* was not reduced by either a combination of 4AP and TEA (K_V_ inhibitors) nor by a combination of cilnidipine and nifedipine (Ca_V_ inhibitors), and the EDTA-induced Na^+^ influx was not reduced by a combination of ω-conotoxin and nifedipine (Ca_V_ inhibitors), suggesting that *I_DF_* is not dependent on K_V_ or Ca_V_. Lastly, inhibition of SOC or TRPM7 (non-selective cation channels that are gated by changes in cytosolic and external divalents) failed to block EDTA-induced responses (Fig. 2B, 8D). It is highly unlikely that sensory neuron-expressed TRPV1, TRPA1 or TRPM8 channels mediate *I_DF_* as these channels are only expressed in a subset (approximately 60%) of vagal neurons, and *I_DF_* was observed in almost all neurons studied. This is supported by the fact that ruthenium red, which also inhibits TRPV1 and TRPA1 [20], failed to prevent EDTA-induced Na^+^ influx.

Reductions in extracellular divalent cations have produced non-selective cation currents in other studies. A reduction of Ca^2+^ evoked transient non-selective cation currents that were inhibited by verapamil but not by external Mg^2+^ in chick DRG neurons [36]. Another study in chick DRG demonstrated a transient voltage-gated non-selective cation current through T-type Ca_V_ that was inhibited by either external Ca^2+^ or Mg^2+^ [5]. Acid-induced (voltage-independent) currents were enhanced by low external divalent cation concentrations in rat vagal neurons [37]. In addition, a substantial persistent voltage-insensitive non-selective cation current was observed upon equimolar replacement of external Ca^2+^ with Mg^2+^ in guinea pig vagal neurons [19]. Besides sensory neurons, the removal of divalent cations has induced non-selective cation currents in myocytes from pig [38] and rat [39] [40], ectodermal cells of chick embryos [41], [42] and Xenopus oocytes [43]. In brief, these currents differ from the present study in that they are either 1) inhibited by L-type or N-type Ca_V_ blockers [36], [38], 2) are transient [5], [36], [38], 3) or are voltage-insensitive [19], [37], [39], [40], [41], [42], [43], or 4) are insensitive to changes in external Mg^2+^, [19], [36].

In summary, we identified a novel voltage-gated, persistent, non-selective cation current in mouse (and guinea pig) vagal sensory neurons that is effectively inhibited by external divalent cations ≥100 µM but is insensitive to blockers of Na_V_, K_V_, Ca_V_, SOC and TRPM7 channels. The physiological relevance of these ion fluxes on vagal neuronal excitability is not known at this time. Extracellular Ca^2+^ and Mg^2+^ concentrations are not fixed in vivo (especially at mucosal-air interfaces) and can be substantially decreased in ischemia [44], [45] and genetic disorders of the TRPM6 gene [46]. Nevertheless we have observed in unclamped neurons substantial Na^+^ influxes as a result of decreased divalent cation external solutions that are capable of triggering voltage-gated Ca^2+^ channel activation, indicating physiologically relevant depolarizations of these neurons.
